# Proteases from *Pleurotus* spp.: Properties, Production and Biotechnological Applications

**DOI:** 10.3390/jof11100702

**Published:** 2025-09-27

**Authors:** Adriane Toledo da Silva, Liliana Aguilar-Marcelino, Amanda do Carmo Alves, Débora Castro Toledo de Souza, Ana Carolina Silva, Jhennifer Cristina de Souza Alves, Yanick Leontino Langa, Elias Honorato Gomes, Filippe Elias de Freitas Soares

**Affiliations:** 1 Laboratório de Biotecnologia e Bioquímica Aplicada, Departamento de Química, Universidade Federal de Lavras, Lavras 37200-900, MG, Brazil; adrianetoledo123@gmail.com (A.T.d.S.); amanda.alves5@estudante.ufla.br (A.d.C.A.); debora.souza7@estudante.ufla.br (D.C.T.d.S.); ana.silva157@estudante.ufla.br (A.C.S.); jhennifer.alves2@estudante.ufla.br (J.C.d.S.A.); yanick.langa@estudante.ufla.br (Y.L.L.); elias.gomes1@estudante.ufla.br (E.H.G.); filippe.soares@ufla.br (F.E.d.F.S.); 2 Centro Nacional de Investigación Disciplinaria en Salud Animal e Inocuidad (CENID-SAI), Instituto Nacional de Investigaciones Forestales, Agrícolas y Pecuarias (INIFAP), Carretera Federal Cuernavaca-Cuautla No. 8534, Colonia Progreso, Jiutepec C.P. 62390, Morelos, Mexico

**Keywords:** edible mushroom, biocontrol, fungi, omics

## Abstract

Proteases (EC 3.4) are hydrolytic enzymes widely used in biotechnological processes, representing about 60 to 70% of the global industrial enzyme market. Edible mushrooms of the genus *Pleurotus* stand out as excellent producers of these enzymes, in addition to exhibiting high nutritional value and medicinal properties. The proteases produced by these species exhibit broad adaptability to different experimental conditions, including variations in optimal pH and temperature, as well as distinct sensitivities to inhibitors. The production of these enzymes can be intensified by solid-state fermentation (SSF) using low-cost agro-industrial substrates, such as wheat bran, which favors sustainable applications aligned with the circular economy. Parameters such as carbon/nitrogen (C/N) ratio, medium pH, cultivation time, and inoculum age directly influence enzyme productivity. Proteases from *Pleurotus* spp. show high potential in the biochemical control of parasites such as *Meloidogyne incognita*, *Haemonchus* spp., *Taenia solium*, and *Moniezia* sp., catalyzing the degradation of the cuticle or eggshell. Other biotechnological applications include milk coagulation, thrombolytic therapies, keratin bioconversion, increased protein digestibility, and use as additives in the food, detergent, and pharmaceutical industries.

## 1. Introduction

Proteases (EC 3.4) are a type of enzyme that helps break down peptide bonds, leading to the formation of peptides and amino acids [1,2,3]. All forms of life contain proteins in their constitution, which play essential roles in fundamental physiological processes such as immune defense, cell growth and death, sporulation, blood coagulation, enzymatic modification, and regulation of gene expression [4,5,6]. These functions confer great biological importance to proteins and, consequently, to proteases. In addition to their physiological relevance, these enzymes occupy a significant share of the industrial market, accounting for approximately 60% to 70% of the enzymes sold worldwide [7,8,9].

Fungi stand out as important microorganisms for protease production [8]. Within this group, edible mushrooms, especially those belonging to the genus *Pleurotus* (phylum Basidiomycota), have gained prominence [10]. The genus *Pleurotus* comprises more than 40 species [4], among which the edible mushrooms *P. ostreatus* (oyster) and *P. eryngii* (king oyster or shimeji) stand out, being widely appreciated and consumed. Globally, *P. ostreatus* is the second most produced mushroom [10,11]. Several factors justify its importance, including medicinal properties, reduced cultivation time compared to other edible mushroom genera, and the ability to colonize and degrade various types of lignocellulosic waste, which favors practices associated with the circular economy [7,9,12].

Proteases of *Pleurotus* spp. have been used promisingly in the biochemical control of different parasites of importance in the context of One Health, such as *Taenia solium*, *Moniezia* sp. [13], *Meloidogyne incognita* [14], *Trichostrongylus* spp., and *Strongyloides papillosus* [15]. They act to catalyze the degradation of proteins present in the structure of the parasites [13]. In addition, these enzymes have potential applications in milk coagulation for cheese production, thrombolytic therapy for the treatment and prevention of thrombosis, and various industrial processes in the detergent, food, and pharmaceutical sectors [3,16,17].

Given the favorable characteristics of proteases from the genus *Pleurotus* and the growing industrial demand for biodegradable methods with low operating costs [18], research involving these fungi has gained increased attention, even though the genera *Aspergillus* and *Penicillium* have historically been the most widely used for protease production [4]. In this context, this review aims to compile up-to-date information on the classification and biochemical characterization of proteases produced by different *Pleurotus* species, as well as discussing their various biotechnological applications.

## 2. Classification and Biochemical Characterization of *Pleurotus* Proteases

The genus *Pleurotus* encompasses a large number of species of protease-producing edible mushrooms [4] such as *P. ostreatus* [11], *P. eryngii* [1], *P. citrinopileatus* [19], *P. pulmonarius* [12], *P. sajor-caju* [18], *P. djamor* [20], *P. albidus* [21], and *P. tuber-regium* [22]. Among these species, the most investigated is *P. ostreatus* [4], due to several factors, such as its high nutritional and medicinal value, excellent yield, ease of large-scale cultivation, and ability to produce a variety of enzymes [4,11].

Among the enzymes produced by species of the genus *Pleurotus*, proteases stand out as one of the most relevant [4,12]. These enzymes can be classified based on their mechanism of action, dividing them into exopeptidases, which promote the catalysis of peptide bond cleavage at the N- or C-terminals, and endopeptidases, responsible for the catalysis of internal bond cleavage in the polypeptide chain [1,23]. In addition, another important classification criterion is the catalytic mechanism involved in hydrolysis, which can be determined by analyzing the amino acid residues or cofactors present at the enzyme’s active site [24,25].

Additionally, proteases can also be classified into families, according to the MEROPS database, which organizes these enzymes based on structural, functional, and evolutionary characteristics. Each family receives an alphanumeric designation, such as S8 (serine proteases) or M36 (metalloproteases/fungalisins) [26].

The most common class of proteases produced by *Pleurotus* spp. is that of serine proteases, whose catalytic triad is composed of aspartate, histidine, and serine residues [9]. During the peptide bond cleavage process, the serine residue acts as a nucleophile, initiating the catalytic reaction. In the industrial context, this type of protease is highly relevant, which explains its great demand on a global scale [9,21]. In MEROPS, these enzymes are mainly assigned to the S8 family. Their activity can be affected by certain inhibitors, such as diisopropyl fluorophosphate (DFP) [27], 3,4-dichloroisocoumarin (DCI), 4-(2-aminoethyl) benzenesulfonyl fluoride (AEBSF), leupeptin, aprotinin, and PMSF (phenylmethylsulfonyl fluoride), the latter being the most cited inhibitor in the literature [2].

On the other hand, although to a lesser extent, aspartic proteases are also produced by *Pleurotus* spp. [28]. These enzymes share several biochemical and structural features, such as sequence similarity, predominance of beta-sheet secondary structures, and the presence of aspartate residues at the active site, which are responsible for catalytic activity [29]. Due to their optimal performance under acidic pH conditions, these enzymes are classified as acid proteases [12]. In MEROPS, they mainly +6 correspond to the A1 family. Inhibitors such as diazoacetylnorleucine (DAN) methyl ester, 1,2-epoxy-3-(p-nitrophenoxy) propane (EPNP), and pepstatin A affect the activity of aspartic proteases, with pepstatin A being the best known and most widely used [2].

Another group of proteases present in the genus Pleurotus is the cysteine proteases, also known as thiol proteases. The active site of these enzymes contains three amino acids that form the catalytic triad Cys/His/Asn (or Asp), whose residues act in a coordinated manner in the hydrolysis of the peptide bond [30]. Although they are not the most frequently found proteases, studies indicate their occurrence in *P. ostreatus*, *P. albidus*, and *P. sajor-caju* [4,21]. In MEROPS, they are grouped into families such as C1. Their activity is inhibited by thiol group-chelating compounds, such as iodoacetic acid [2].

Metalloproteases are also present in *Pleurotus* species, such as *P. ostreatus* and *P. eryngii* [17,31]. The enzymatic catalysis of these proteases requires the coordination of a metal ion at the active site. During peptide bond hydrolysis, the water molecule, acting as a nucleophile, is activated by the metal ion [32]. In MEROPS, metalloproteases of the M36 family, also known as fungalisins, are particularly noteworthy and are frequently found in basidiomycetes. The activity of these enzymes is inhibited by metal-chelating agents, such as EDTA (ethylenediaminetetraacetic acid) and 1,10-phenanthroline [2,29].

The presence of different classes of proteases may be associated with the different morphological stages of edible mushrooms. In the study by Genier et al. (2015), for example, the authors suggested that the protease produced by *P. ostreatus* is a serine protease, based on the total inhibition of enzymatic activity observed in the presence of a specific inhibitor of this class [33]. On the other hand, Choi and Shin (1998), using the fruiting body as the enzyme source in the purification and characterization stage, identified a cysteine protease produced by *P. ostreatus* [34].

In genomic terms, the genome of the PC9_AS strain of *P. ostreatus* was annotated with approximately 11,875 genes, a number similar to that found in strains PC15 and PC9_JGI, which have 12,330 and 12,206 genes, respectively [35]. The annotation includes, among other elements, protease-encoding genes classified by the MEROPS database, suggesting considerable diversity of these enzymes in the genome. Transcriptomic studies reinforce this enzymatic diversity: Alfaro et al. (2016) identified at least 18 peptidase families, including serine, cysteine, aspartic, and metalloproteases, with emphasis on the S8 (serine proteases) and M36 (metalloproteases) families, which showed high expression under different culture conditions [36]. In addition, Faraco et al. (2005) characterized a new subfamily of serine proteases in *P. ostreatus*, describing the structure and function of an extracellular protease encoded by the posl gene, belonging to the S8 family, confirming its relevant role in the degradation of lignocellulosic substrates [37]. The review by Inácio et al. (2015) highlights that the genus *Pleurotus* possesses a variety of extracellular proteases, reflecting an adaptive strategy that contributes to its efficiency in decomposing organic matter, especially in environments rich in lignin and cellulose [4]. However, despite the identification of multiple peptidase families and the functional annotation of the genome, to date there are no studies that present, in a consolidated manner, the total number of protease-encoding genes in the genus *Pleurotus*, which remains a gap in the literature.

Table 1 presents the main biochemical properties of proteases produced by various *Pleurotus* species, including molecular weight, optimal pH and temperature, sensitivity to inhibitors, and response to metal ions. These parameters can vary significantly between and within species due to differences in the media used to induce enzyme production, which highlights the functional diversity of fungal proteases.

When analyzing Table 1, the wide adaptability of *Pleurotus* spp. proteases to environmental conditions can be observed. These enzymes show significant variations in their optimal pH and temperature ranges [2,23]. In addition, the use of specific inhibitors for different classes of proteases, such as PMSF, pepstatin A, and EDTA, has revealed the presence of multiple classes of proteases in the same species [2,5]. The relevance of metal ions in enzymatic catalysis further reinforces the importance of detailed characterization of these enzymes, especially for their application in biotechnological processes involving different matrices and physicochemical conditions [5,12].

## 3. Physiological Aspects of Protease Production by *Pleurotus* spp.

The presence of different classes of proteases may be associated with the different morphological stages of the edible mushrooms. In the study by Genier et al. (2015), for example, the authors suggested that the protease produced by *P. ostreatus* is a serine pro-tease, based on the total inhibition of enzymatic activity observed in the presence of a specific inhibitor of this class [33]. On the other hand, Choi and Shin (1998), using the fruiting body as an enzyme source in the purification and characterization stage, identified a cysteine protease produced by *P. ostreatus* [34].

The age of the inoculum is a critical and easily controlled factor that directly impacts protease production yield [4]. Previous studies indicate that a young inoculum in an active growth phase results in significantly higher enzyme production [21]. The study by Martim et al. (2017) [21] using *P. albidus* found maximum activity (80.33 U/mL) with a five-day-old inoculum. Subsequently, the authors observed a gradual decrease in activity with increasing inoculum age, with reductions of 28.57%, 47.40%, and 77.39% in proteolytic activities for inocula aged 8, 12, and 20 days, respectively [21].

The cultivation time determines the peak production of both biomass and enzymes, varying significantly among *Pleurotus* spp. species (*P. sajor-caju* at 4 days, *P. djamor* at 5 days, and *P. pulmonarius* at 10 days) [4,45]. Exceeding the ideal time results in a decrease in enzymatic activity, possibly due to enzymatic degradation or toxin accumulation [7,24].

A key variable that controls both microbial growth and protease production for *Pleurotus* spp. is the C/N ratio in the substrate [45]. Adequate amounts of carbon, particularly from lignocellulosic sources such as wheat, stimulate the development of the fungus, as protease production is often inhibited by a lack of carbon and nitrogen, forcing the fungus to secrete these enzymes to obtain nutrients from the medium. On the other hand, an excess of nitrogen can be toxic and inhibit mycelial growth [7].

In addition to hydrolyzing proteins, *Pleurotus* spp. proteases play an important role in regulating other enzymes produced by the fungus. It has been demonstrated that the extracellular acid and neutral proteases produced by *P.* ostreatus modulate the activity of laccase isoforms by degrading enzymes such as POXA1b via the serine protease PoSl [2,18]. Conversely, when these proteases are inhibited by inhibitors such as PMSF and pepstatin A, laccase activity increases by a factor of 1.35 [2,37].

In contrast, alkaline proteases have been shown to have a low or even positive effect on *P. ostreatus* activity. This indicates that laccase is not sensitive to this type of enzyme and can therefore act as an activator of laccase activity [4,10]. The literature also highlights possible synergistic or competitive interactions between proteases and laccases influenced by substrate composition, revealing a highly adaptive enzyme system [2].

At the physiological level, these enzymes act in spore germination, sporulation, and substrate degradation, being fundamental for the survival of the fungus in lignocellulosic environments [2,4]. The co-expression of proteases and laccases produced by *P. sapidus* suggests the presence of integrated enzyme systems, although these are still poorly understood [11].

## 4. Production and Optimization of *Pleurotus* Proteases

The production of enzymes by mushrooms of the genus *Pleurotus* mainly uses two different strategies: solid-state fermentation (SSF) and submerged fermentation (SmF) [7,24]. These fungi are significant producers of various enzymes, among which proteases stand out for their extensive industrial applications. Therefore, selecting an appropriate cultivation method is essential to increase enzyme production and economic viability [9,19].

Solid-state fermentation (SSF) mimics the natural habitat of many fungi, cultivating microorganisms in isolated or low-moisture substrates [47]. Because it uses less water and energy and produces less harmful waste, this process is believed to be more cost-effective [48]. On the other hand, submerged fermentation (SmF) (Table 2) involves the cultivation of microorganisms in a liquid medium. Immersing the fungus provides greater control over culture parameters such as pH, temperature, oxygen levels, and humidity, in addition to simplifying the purification of extracellular enzymes and large-scale production [21,49,50].

The choice between SSF and SmF requires a strategic assessment of the various possibilities and production interests. On the one hand, SSF is commonly chosen because it generates a more concentrated enzyme product, which facilitates and reduces the costs of the extraction and purification phases, making it attractive in some biocatalysis processes [12]. However, despite higher initial costs, SmF offers better process control and greater scalability, essential factors for consistent industrial production. Both approaches are being actively investigated to maximize protein yield for the growing biotechnology market [2,20].

In general, the success of this species in bioprocesses is due to its ability to grow easily in tropical and subtropical regions. *Pleurotus* spp. species are especially suitable for SSF because they can thrive on a variety of lignocellulosic agricultural wastes and are mainly capable of mimicking their natural growth environment, as shown in Table 3 [52].

The proteases produced by fungi of the *Pleurotus* genus show highly competitive enzymatic activity, with values that often exceed those of other fungi in specific applications, although direct comparison requires methodological caution [24]. An example of this is the fibrinolytic activity of the *P. eryngii* species, which showed larger activity halos (63 mm^2^) than *Agaricus blazei* (35 mm^2^) and a specific activity of 226.47 U/mL, higher than that of *P. ostreatus* (71.5 U/mL) [6,17]. Even more significantly, the serine protease produced by *P. sajor-caju* showed a catalytic efficiency up to 8.48 times greater than that of *Aspergillus oryzae* [18]. On the other hand, in some cases it is possible to observe that genera that belong to the Ascomycota phylum excelled in proteolytic activity, such as *Duddingtonia flagrans* (56.34 U/mL) [53], *Beauveria bassiana* ESALQ PL63, and *Metarhizium anisopliae* ESALQ E9 (36 U/mg and 52 U/mg), respectively [54]. During the milk curdling process, the gross activity of *P. albidus* (153 U/mL) remained lower than that of *Aspergillus* sp. (240 U/mL) [5]. It is important to note that these values are dependent on the substrate and test conditions, but the consolidated evidence positions *Pleurotus* as a versatile and potent source of proteases with high biotechnological potential [4].

The efficiency of the *Pleurotus* genus in degrading lignocellulosic substrates is what underpins its use in circular economy applications, such as the production of proteases from agro-industrial waste [55,56]. As decomposition is necessary for its growth, the fungus actively seeks out these materials, which are rich in cellulose, hemicellulose, and lignin [57]. These fungi are able to carry out this process because they produce a complex of ligninolytic and hydrolytic enzymes, including proteases, which act synergistically to catalyze the breakdown of vegetative cell wall polymers [52,58]. Thus, while the fungus performs its ecological function of recycling nutrients, it also produces high-value biotechnological enzymes, converting waste into a product [3,59].

For the enzymatic production process to transcend the laboratory scale and become an industrially viable process, it is important to rigorously optimize the cultivation parameters [51]. Variables such as pH, temperature, incubation time, agitation, and the nutritional composition of the substrate, particularly the carbon/nitrogen (C/N) ratio, interact synergistically to modulate the fungus’s metabolism and directly affect its production process. Precise control of these variables is what determines the commercial success of the bioprocess, maximizing productivity, reducing operating costs, and ensuring the acquisition of enzymes with the desired activity and stability [48].

The pH of the culture medium influences the activity, production, and stability of *Pleurotus* spp. proteases [23]. Each species and even each type of protease may have a distinct optimum pH (e.g., pH 6.5 for *P. eryngii*, pH 9.0 for *P. ostreatus* alkaline) [1,33]. The pH of the medium is also dynamic, being influenced by factors such as the C/N ratio, and its optimization is vital not only for production but also for specific applications, such as milk coagulation in cheese making [23].

## 5. Applications of *Pleurotus* Proteases

### 5.1. Nematicide Applications and Biological Control

In addition to their importance in human nutrition and biotechnological processes, mushrooms of the *Pleurotus* genus have attracted growing interest for their potential in the biological control of animal parasites such as *Moniezia* sp., *T. solium*, and *Haemonchus* spp. and plant parasites such as *M. incognita* [13,14,15,46]. The use of *Pleurotus* spp. as biocontrol agents represents a promising alternative to reduce dependence on chemical pesticides, contributing to more sustainable and environmentally friendly agricultural practices [11].

These fungal species act through different mechanisms to control parasites, one of which is the production of toxins, such as trans-2-decenedioic acid, capable of paralyzing target organisms. Once paralyzed, the parasites become entangled in the fungal hyphae and exhibit morphological deformities, such as a reduction in head volume [60,61].

**Figure 1:**
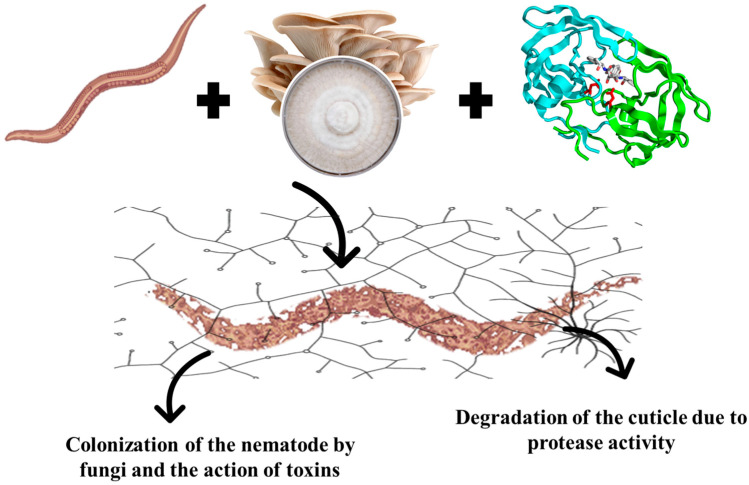
Mechanism of nematophagy of edible mushroom.

Another important mechanism involves the secretion of extracellular proteases. The cuticle of nematodes, composed predominantly of proteins, functions as a protective barrier. Proteases produced by *Pleurotus* spp. act directly on this structure, catalyzing the hydrolysis of cuticular proteins, leading to its degradation and, consequently, to the death of the parasite [14] (Figure 1).

In vitro studies have used free-living nematodes, such as *Panagrellus* sp., as models to evaluate the efficacy of compounds produced by *Pleurotus* spp. Genier et al. (2015) [33] evaluated the predatory activity of *P. ostreatus* (strain PLO 06) on *Panagrellus* sp. larvae, observing a 65.6% reduction in the number of larvae on the first day, 77.4% on the second day, and 95.2% on the third day. In the same study, in another trial, only the extracellular proteases from the same isolate were used, resulting in a 42% reduction in the number of larvae. This result indicates that the proteases produced by *P. ostreatus* (90 U/mL) are essential for the cuticle degradation process, enhancing the nematicidal effect [33].

The combined action of toxins that paralyze nematodes and hydrolytic enzymes contributes to the biocidal effect promoted by species of the genus *Pleurotus.* Plant parasitic nematodes cause great damage to agriculture worldwide, directly affecting crop yields and requiring more effective control strategies [14]. In this context, Sufiate et al. (2017) demonstrated that *P. eryngii* has nematicidal activity on eggs and juveniles of *M. javanica*, with a reduction of more than 50%, attributed to the production of proteases (32.74 U/mL) and chitinases (3.57 U/mL) [62].

Tests conducted with animal parasitic nematodes also demonstrate the nematicidal potential of *Pleurotus* spp. proteases. Silva et al. (2025) evaluated the effect of the cell-free crude extract, rich in *P. djamor* proteases (proteolytic activity of 7.5 U/mL and specific activity of 30 U/mL), on ruminant coprocultures and observed a 35% reduction in the number of *Haemonchus* spp. and *Trichostrongylus* spp. larvae, a result considered promising, especially as it was obtained in a fecal environment [46].

In addition to nematodes, in vitro tests on *T. solium* and *Moniezia* sp. eggs demonstrated the potential of *P. djamor* proteases (31.61 U/mL) in controlling these parasites. Silva et al. (2025) reported reductions of 33.44% in the hatching of *T. solium* eggs and 45.43% in *Moniezia* sp. eggs, highlighting the role of proteases in the degradation of eggshells [13].

The synergistic action between toxins and enzymes intensifies the observed effects. Trans-2-decenedioic acid, for example, initially acts by paralyzing the parasites, compromising their ability to respond and making them more susceptible to enzymatic action [20]. In turn, the degradation of the cuticle promoted by proteases facilitates toxin penetration, establishing an effective cycle of action. This synergy gives *Pleurotus* spp. a significant advantage as biocontrol agents [33].

The use of *Pleurotus* spp. or its cell-free crude extracts has several ecological advantages. However, further advances are still needed in the standardization of fermentation processes, purification of bioactive compounds, and more in-depth field studies to assess enzyme stability [11]. *Pleurotus* spp. and their proteases thus prove to be a promising biotechnological tool in controlling different parasites. The combined action of toxic metabolites and extracellular proteases confers high efficacy in the immobilization and degradation of these organisms. Case studies with different species of the genus [33,62] reinforce this potential, indicating the possibility of developing effective and sustainable bio-inputs for modern agriculture.

### 5.2. Food

Cheese production reflects the cultural and culinary traditions of each country and region. In 2019, the global cheese market was valued at approximately US$114.1 billion. Europe is the leading producer, accounting for nearly half of the total market value. In terms of consumption, the United States ranks first, followed by Germany and France. It is estimated that by the end of 2030, global cheese production will reach 27 million tons, underscoring the relevance of cheese as a key product in the food sector [63].

An essential step in cheese production is milk coagulation. This process occurs through the hydrolysis of peptide bonds in κ-casein, a protein present in milk, which leads to micelle destabilization and, consequently, the precipitation of casein [42]. One of the main methods used for inducing milk coagulation involves the action of hydrolytic enzymes, with chymosin (EC 3.4.23.4) being the most widely employed. This protease is considered the standard enzyme for milk coagulation in the production of various types of cheese due to characteristics such as high coagulating activity and specificity for cleaving the Phe105–Met106 peptide bond in κ-casein [23].

Renin, a complex of aspartic endopeptidases, is the main source of proteases used in cheese production. However, the use of raw materials of animal origin, such as bovine stomachs, has prompted the search for alternative sources of milk-coagulating enzymes. Species of the *Pleurotus* genus have a remarkable ability to adapt to different environments, since they are able to colonize and degrade a wide variety of lignocellulosic residues, in addition to growing in different temperature ranges and geographical regions [18,33]. It is one of the most widely distributed genera of edible mushrooms in the world, and its cultivation is facilitated by its low nutritional requirements [4,18]. In addition, they produce high levels of various enzymes, particularly proteases that are essential for the hydrolysis of milk proteins, making them promising alternatives as sources of enzymes for cheese production [18,21,42].

Studies have shown that one species of this genus, *P. albidus*, native to the Amazon, is capable of synthesizing milk-coagulating proteases in liquid culture media. Martim et al. (2021) [23] demonstrated that the proteases produced by *P. albidus* (DPUA 1692), when cultivated on açaí seeds supplemented with rice bran, were effective in the production of *Minas frescal* cheese. Fermentation time and temperature, as well as the concentration of certain metal ions such as calcium, influenced their coagulating activity [23].

A previous study by Martim et al. (2017) also reported that *P. albidus* (DPUA 1692) produces milk-coagulating enzymes with optimal activity at 60 °C and pH 6 [21]. Nolli et al. (2022) observed that the crude extract of *P. albidus* with an activity value of 153 U/mL was sufficient to coagulate milk [5].

In addition, another edible mushroom species that has shown the ability to produce milk-coagulating proteases is *P. djamor*. Research conducted by Silva et al. (2025) [9] reported the production of these enzymes by *P. djamor* PLO13 cultivated in wheat bran, identifying them as serine proteases. These enzymes were capable of coagulating both whole and skimmed pasteurized milk, even at room temperature and in the presence or absence of calcium. According to the authors, 1.875 mg/mL of protease-containing protein in the crude extract of *P. djamor* is required to coagulate milk at a temperature of 50 °C in 30 min for pasteurized whole milk and in 45 min for reconstituted skimmed milk [9].

### 5.3. Biomedical

According to an article published by Mensah et al. (2023), the global death toll from cardiovascular disease rose from 12.4 million in 1990 to 19.8 million in 2022, reflecting the growth and aging of the world’s population, as well as the influence of preventable risk factors of a metabolic, behavioral, and environmental nature [64]. In addition, although the available drugs are safe, they are still expensive [65]. In this context, there is evidence that enzymes produced by microorganisms, such as fungi, may be useful in controlling cardiovascular diseases due to their ability to produce fibrinolytic enzymes [24].

Fibrinolytic enzymes are proteases capable of promoting the catalysis of the degradation of the fibrin mesh, the main protein component present in blood clots. Among the species capable of producing fibrinolytic enzymes are mushrooms of the genus *Pleurotus*, such as *P. ferulae*, *P. ostreatus*, *P. eryngii*, and *P. pulmonarius* (Table 4). The fibrinolytic proteases produced by these fungi have been identified as SPPs, metalloproteases, serine proteases, and serine metalloproteases, showing therapeutic potential against thrombosis [4,17].

### 5.4. Industrial

Keratinases are extracellular proteases capable of catalyzing the degradation of insoluble keratin-rich substrates such as feathers, hair, and nails, which are highly stable structures due to their high content of disulfide bonds, making them resistant to most common proteases [4,19,37]. These enzymes, generally classified as serine or metalloproteases, have been investigated in ligninolytic fungi such as *Pleurotus* spp., whose enzymatic diversity reveals applications in sustainable processes [2,4,10].

The products of keratin hydrolysis are mainly amino acids and smaller peptides. These compounds can be applied in various sectors, such as in the production of fertilizers, in the pharmaceutical and food industries, in environmental pollution control, and in leather processing, among others [10,43].

For example, in *P. pulmonarius*, a 16 kDa protease with proven keratinolytic activity on feathers, hair, and human keratin, both in vitro and in vivo, was identified, indicating its value in the bioconversion of keratinous waste [4,43]. Its effectiveness has also been demonstrated in lignocellulosic substrates, extending its scope beyond keratins [12]. In addition, enzymes from *P. ostreatus* and *P. sapidus* have been associated with the degradation of structural proteins in lignin-rich environments, which highlights the adaptive role of these proteases in various biotechnological systems [2,11,37].

The application of *Pleurotus* spp. proteases in protein hydrolysis has proven effective in increasing digestibility and generating bioactive peptides with functional potential. Alkaline proteases obtained from *P. eryngii* were applied in the pretreatment and promoted an increase of up to 7.54 times in the release of amino acids, with emphasis on the L isomers of lysine and arginine, in addition to the generation of up to 19,870 short peptides (<800 Da), which are highly bioavailable. This result is noteworthy because the amino acids mentioned are important in the context of human health and muscle recovery. Making their bioavailability more efficient contributes to their nutritional value [10].

Proteases from *Pleurotus* spp. are promising biocatalysts for industrial applications, given their specificity, efficiency, and biodegradability [4,19]. They are used in detergents, food, leather, silk, cosmetics, wastewater treatment, and metal recovery, such as silver. Their stability in alkaline pH favors their use as additives for detergents [37].

In this context, subtilisin PPP1 from *P. pulmonarius* has proven effective in removing organic stains, while the thermostable protease SPPS, isolated from *P. sajor-caju*, performs well under industrial conditions [18,66]. Serine proteases, PoSl, are widely researched and dominate the industrial enzyme market [67]. In addition, native strains such as *P. albidus*, from the Amazon, produce high levels of proteases (34.00 U/mL), highlighting the regional value of these species [21].

## 6. Conclusions and Future Prospects

The *Pleurotus* genus of fungi stands out as an important producer of proteases, whose biochemical properties offer broad potential for applications in areas such as medicine, agriculture, the food industry, and biotechnology. In addition to their functional diversity, these enzymes exhibit promising nematicidal potential and can be obtained from agro-industrial residual substrates, providing environmental and economic benefits by adding value to materials that would otherwise be discarded.

Despite recent advances, significant gaps remain in the understanding of the metabolic and molecular processes involved in the production of these enzymes, making it essential to explore new biotechnological strategies to enhance their efficiency and stability. Modern approaches, such as the immobilization of cells or enzymes on innovative supports, including nanoparticles, mesoporous materials, hydrogels, and structures derived from agro-industrial residues, have shown promising results by increasing catalytic activity, resistance to adverse conditions, and the potential for reuse in industrial processes.

In parallel, genetic engineering techniques offer pathways for obtaining more robust variants with higher yield and specificity, including in recombinant hosts, thereby expanding prospects for large-scale production. The growing demand for proteases with high stability, specificity, and sustainability creates a favorable environment for the development of innovative solutions based on *Pleurotus* spp.

*Pleurotus* proteases exhibit promising characteristics, including stability under alkaline conditions and high catalytic activity in challenging environments. These attributes enhance their potential for industrial, agricultural, and biomedical applications, highlighting their biotechnological value and reinforcing their potential as sustainable and competitive alternatives to existing market solutions.

Thus, the development of enzyme formulations derived from *Pleurotus* spp. for biochemical pest control, industrial processes, and high-precision biomedical applications represents a strategic pathway for innovation. Continuous investment in scientific research and technological development is essential to establish *Pleurotus* proteases as sustainable, innovative alternatives with economic and social relevance, promoting solutions aligned with current trends in biotechnology.

## Figures and Tables

**Table 1:** Biochemical properties of proteases from species of the genus *Pleurotus*.

Species	Molecular Weight (kDa)	Optimal pH	Optimal Temperature (°C)	Km	Vmáx	Sensitivity to Inhibitors	Influence of Metal ions on Enzymatic Activity
*P. eryngii*	11.5 [38] 14 [29]	5.0 [38] 6.5 [1]	40 [27]	0.18 mM [10]	53.5 U/mL [10]	Pepstatin A inhibits pleureryn; PMSF does not affect it [38]. The fibrinolytic enzyme was completely inhibited by PMSF [27]	-
*P. citrinopileatus*	28 [19]	10 [19]	50 [19]	3.44 mg/mL [19]	0.139 mg/ mL·min [19]	PMSF completely inhibited, EDTA did not inhibit, Pepstatin A moderately inhibited [19]	Activation by K^+^, Li^+^, and Mg^2+^; inhibition by Al^3+^, Cu^2+^, Hg^2+^, Zn^2+^, Pb^2+^, Co^2+^, Mn^2+^, and Ca^2+^ [19]
*P. ostreatus*	18.2–97 [24,28,29,34,39,40,41]	6.5–9 [20,33,39,42]	35–75 [33,34,41,42]	-	-	PMSF completely inhibited [2,32,39], Pepstatin A inhibits acid protease [2], *P. ostreatus* subtilisin-like protease was not inhibited by Pepstatin A [39], EDTA inhibited it completely [33], and protease was inhibited by EDTA only for a certain period of time to 74.3% [2]	Ca^2+^ has a positive effect on activity [39]. Cu^2+^, Al^3+^, and Hg^2+^ cause inhibition. Activity was restored by Zn^2+^ or Co^2+^ [19]
*P. pulmonarius*	16 [43]	5.5 [12]	45 [12]	0.61 mg/mL	1.79 mM/min	Pepstatin A (56% inhibition), PMSF (63.4% inhibition), EDTA (81.0% maximum inhibition) [12]	Inhibition by Mn^2+^, Ba^2+^, Fe^2+^, and Ca^2+^. Slight increase by Mg^2+^ [12]
*P. albidus*	36 [5]	7.0 [5] 5.0 [23]	40–50 [5,23]	-	-	PMSF (30% inhibition) [23]. Pepstatin A and EDTA did not inhibit [21]	Mn^2+^, Cu^2+^, Na^+^, Mg^2+^, K^+^, Zn^2+^, and Ca^2+^ cause slight inhibition. Fe^2+^ increased activity [21,23]
*P. ostreatoroseus*	-	7.0 [44]	40 [44]	-	-	PMSF (94% inhibition). EDTA (87% inhibition) [21]	Cu^2+^, Zn^2+^, and Mn^2+^ cause inhibition, while Fe^2+^, Mg^2+^, Ca^2+^, K^+^, and Na^+^ cause slight inhibition [21]
*P. sajor-caju*	48 and 65 [18,45]	8.0 [45]	60 [45]	0.275 mg/mL	79 μmol/ mg/min	PMSF completely inhibited, EDTA slightly inhibited, and pepstatin A did not inhibit [18]	Cd^2+^, Ni^2+^, Hg^2+^ were inhibited completely. Co^2+^, Ba^2+^, Zn^2+^ inhibited partially. Activation by Ca^2+^, Mg^2+^, Mn^2+^, and Fe^2+^ increased activity [18]
*P. djamor*	75–100 [46]	5.0 and 8.0 [9,20]	50 [20]	-	-	PMSF (45% inhibition) and EDTA (69% inhibition) [9]	-

**Table 2:** Submerged fermentation (SmF) for protease production by *Pleurotus* spp. species.

Agro-Industrial Residues	*Pleurotus* Species	Proteolytic Activity (U/mL)	Study
Glucose, yeast extract	*P. ostreatus*	90.0	[33]
Glucose, yeast Extract, peptone	*P. albidus*	73.39	[21]
Potato dextrose broth	*P. sajor-caju*	10.5	[18]
Yeast Malt	*P. ostreatus*	1015.1	[17]
Yeast Malt	*P. eryngii*	1552.8	[17]
Sabouraud dextrose, yeast extract	*P. ostreatus*	1512.0	[17]
Sabouraud dextrose, yeast extract	*P. eryngii*	1086.4	[17]
Glucose, peptone, yeast extract	*P. ostreatoroseus*	1361.73	[51]
Malt, glucose, peptone, yeast extract	*P. ostreatoroseus*	262.02	[51]
Malt	*P. ostreatoroseus*	382.32	[51]

**Table 3:** Lignocellulosic and agroindustrial residues used as alternative substrates for protease production by *Pleurotus* spp. species.

Agro-Industrial Residues	*Pleurotus* Species	Proteolytic Activity (U/mL)	Study
Wheat Bran	*P. albidus*	5.029	[5]
Wheat Bran	*P. djamor*	31.61	[13]
Wheat Bran	*P. pulmonarius*	76.3	[12]
Wheat Bran	*P. sajor-caju*	22	[45]
wheat grains	*P. ostreatus* (SB)	5.0	[20]
wheat grains	*P. ostreatus (Pearl)*	4.0	[20]
Oat bran	*P. pulmonarius*	73.0	[12]
Corn Cob	*P. eryngii*	53.5	[28]
Corn Flour	*P. sajor-caju*	45	[45]
Corn pomace	*P. pulmonarius*	94.0	[12]

**Table 4:** Studies of fungi of the genus *Pleurotus* that showed fibrinolytic enzyme production and activity.

*Pleurotus* Species	Cultivation Method	Specific Activity	Fibrinolytic Activity	Evaluation Method	Study
*P. ostreatus*	Fermentation in submerged culture	1.199, 75 U/mg	-	Fibrin plate method	[49]
*P. ostreatus*	Soybean Bran + Yeast Extract	-	100.14 U/mL	Formation of an artificial thrombus	[17]
*P. ostreatus*	Yeast Extract Malt broth	-	71.5 ± 0.56 U/mL	Formation of an artificial thrombus	[17]
*P. eryngii*	Yeast Extract Malt broth	-	226.47 U/mL	Formation of an artificial thrombus	[17]
*P. eryngii*	Soybean Bran + Yeast Extract	-	71.49 U/mL	Formation of an artificial thrombus	[17]
*P. albidus*	Solid state fermentation (Black-eyed peas)	850 U/mg	181.11 U/mL	Fibrin plate method	[65]
*P. albidus*	Solid state fermentation (Thorn yam)	310 U/mg	156.01 U/mL	Fibrin plate method	[65]
*P. albidus*	Solid state fermentation (Wheat grain)	180 U/mg	128.40 U/mL	Fibrin plate method	[65]
*P. ostreatus*	Malt extract agar	6.45 mm^2^	-	Fibrin plate method	[3]
*P. ferulae*	-	1.253, 33 U/mg	376 U/mL	Fibrin plate method	[31]

## Data Availability

No new data were created or analyzed in this study. Data sharing is not applicable to this article.
